# Severe Cardiovascular Sequelae in Adults After Kawasaki Disease

**DOI:** 10.1001/jamanetworkopen.2025.26396

**Published:** 2025-08-12

**Authors:** Yoshihide Mitani, Michikazu Nakai, Etsuko Tsuda, Toshihiro Tamura, Yasutsugu Shiono, Hiroyoshi Yokoi, Hiroyuki Ohashi, Hirofumi Sawada, Masahiro Hirayama

**Affiliations:** 1 Department of Pediatrics, Mie University Graduate School of Medicine, Mie, Japan; 2 Clinical Research Support Center, University of Miyazaki Hospital, Miyazaki, Japan; 3 Department of Pediatric Cardiology, National Cerebral and Cardiovascular Center; 4 Department of Cardiology, Tenri Hospital; 5 Department of Cardiovascular Medicine, Wakayama Medical University, Wakayama, Japan; 6 Department of Cardiology, International University of Health and Welfare

## Abstract

This cohort study evaluates the incidence and outcomes of cardiovascular hospitalizations among adults in Japan with a history of Kawasaki disease.

## Introduction

Kawasaki disease (KD), first described in 1967, is an acute childhood vasculitis that can have lifelong coronary sequelae.^1,2,3^ Although most children now receive timely therapy, severe coronary aneurysms still place affected patients at risk for serious long-term cardiac complications. Patients with large coronary aneurysms may experience acute myocardial infarction shortly after the acute phase or remain asymptomatic for decades, only developing ischemic events and requiring revascularization in adulthood.^1,2,3,4,5^ With the earliest KD cohorts now reaching their 30s and 40s, clinicians increasingly encounter late cardiovascular complications, including acute coronary syndrome (ACS), chronic coronary syndrome, ischemic cardiomyopathy, heart failure, and arrhythmias. Nonetheless, robust epidemiological data and clear prognostic factors for severe adult cardiovascular sequelae remain scarce, particularly concerning the continuity of care from childhood into adulthood.

## Methods

This cohort study was approved by the Ethics Committees of Mie University Hospital and the National Cerebral and Cardiovascular Center, with a waiver of informed consent. Reporting followed the STROBE guideline. Additional methods are detailed in the eMethods in Supplement 1. We conducted a retrospective cohort analysis using data from the Japanese Registry of All Cardiac and Vascular Diseases–Diagnosis Procedure Combination (JROAD-DPC), which compiles clinical records from cardiovascular hospitals nationwide in Japan.^6^ We included adult patients (≥15 years) hospitalized between April 2013 and March 2022 with severe KD-related cardiovascular events who were identified using the *International Statistical Classification of Diseases and Related Health Problems, Tenth Revision *(*ICD-10*). Emergency and nonreferral admissions served as proxies for disrupted continuity of care. Outcomes were in-hospital death (primary) and intensive care unit (ICU) admission (secondary). Multilevel mixed-effects logistic regression (random intercept for hospital) generated odds ratios (ORs) with 95% CIs for in-hospital mortality or ICU admission using 2 models: model 1, age, sex, and emergency admission; and model 2, age, sex, and nonreferral admission. Stata version 16.1 (StataCorp) was used. Statistical significance was set at a 2-sided *P* < .05.

## Results

Our analysis included 798 hospitalizations (median [IQR] age, 37 [23-44] years; 74.4% male). Clinical presentations were categorized as ACS (19.7%), percutaneous coronary intervention (13.0%), coronary artery bypass grafting (14.2%), and heart failure or arrhythmia (53.1%) (Table). The median (IQR) BMI (calculated as weight in kilograms divided by height in meters squared) was 22.9 (20.3-25.6); 197 patients (24.7%) currently smoked, rising to 59 of 157 (37.6%) among those with ACS. At discharge, 71.4% received antiplatelet therapy (81.5% among ACS cases), 25.2% received systemic anticoagulation, and just 31.1% were started on a statin. Most admissions occurred at teaching hospitals (92.2%). Emergency and nonreferral admissions accounted for 33.0% and 16.0% of admissions, respectively. The overall ICU admission was 27.6%, and in-hospital mortality was 1.3%. Age distribution was bimodal, peaking at younger than 20 years and between the ages of 35 and 39 years, whereas ACS showed only the older peak (Figure). Multivariable analysis revealed that emergency admissions (OR, 8.49; 95% CI, 1.80-40.04; *P* = .007) and nonreferral admissions (OR, 6.69; 95% CI, 1.68-26.60; *P* = .007) were associated with increased mortality risk and that nonreferral admission (OR, 1.73; 95% CI, 1.08-2.78; *P* = .02) was associated with increased risk of ICU admission.

**Table.** Demographic Characteristics

Characteristic	Participants, No. (%)				
	Overall (N = 798)	ACS (n = 157)	PCI (n = 104)	CABG (n = 113)	HF or arrhythmia (n = 424)
Age, y					
No. with available data	798	157	104	113	424
Median (IQR)	37.0 (23.0-46.0)	39.0 (32.0-47.0)	38.0 (27.0-46.0)	39.0 (29.0-45.0)	34.0 (20.0-45.0)
Sex					
Male	594 (74.4)	124 (79.0)	79 (76.0)	92 (81.4)	299 (70.5)
Female	204 (25.6)	33 (21.0)	25 (24.0)	21 (18.6)	125 (29.5)
BMI					
No. with available data	777	150	103	113	412
Median (IQR)	22.9 (20.3-25.6)	24.4 (20.9-26.9)	23.1 (21.1-26.8)	23.5 (20.8-26.1)	22.1 (19.8-25.1)
Current smoking	197 (24.7)	59 (37.6)	31 (29.8)	27 (23.9)	80 (18.9)
Treatment					
HF or arrhythmia					
Any	718 (90.0)	117 (74.5)	64 (61.5)	113 (100)	24 (100)
HF	474 (59.4)	101 (64.3)	44 (42.3)	113 (100)	216 (50.9)
Arrhythmia	535 (67.0)	89 (56.7)	49 (47.1)	109 (96.5)	288 (67.9)
PCI					
Any	191 (23.9)	85 (54.1)	104 (100)	2 (1.8)	0
POBA	73 (9.1)	41 (26.1)	31 (29.8)	1 (0.9)	0
Stenting	73 (9.1)	36 (22.9)	37 (35.6)	0	0
PTCRA	38 (4.8)	3 (1.9)	34 (32.7)	1 (0.9)	0
Intracoronary thrombolysis	2 (0.3)	1 (0.6)	1 (1.0)	0	0
Aspiration thrombectomy	13 (1.6)	13 (8.3)	0	0	0
CABG	148 (18.4)	34 (21.7)	0	113 (100)	0
Ablation	31 (3.9)	0 (0)	2 (1.9)	0	29 (6.8)
IABP	54 (6.8)	42 (26.8)	2 (1.9)	7 (6.2)	3 (0.2)
PCPS	11 (1.4)	8 (5.1)	0	1 (0.9)	2 (0.5)
Oral medication at discharge					
Antihypertension	399 (50.0)	97 (61.8)	43 (41.3)	92 (81.4)	167 (39.4)
Antidiabetes	17 (2.1)	3 (1.9)	3 (2.9)	2 (1.8)	9 (2.1)
Anticoagulant					
Any	201 (25.2)	62 (39.5) 1	2 (11.5)	34 (30.1)	93 (21.9)
Warfarin	173 (21.7)	57 (36.4)	9 (8.7)	29 (25.7)	78 (18.4)
DOAC or NOAC	28 (3.5)	5 (3.2)	3 (2.9)	5 (4.4)	15 (3.5)
Antiplatelet					
Any	570 (71.4)	128 (81.5)	90 (86.5)	104 (92.0)	248 (58.5)
Aspirin	454 (56.9)	109 (69.4)	64 (61.5)	98 (86.7)	183 (43.2)
Other antiplatelet	351 (44.0)	85 (54.1)	86 (82.7)	45 (39.8)	135 (31.8)
DAPT	235 (29.4)	66 (42.0)	60 (57.7)	39 (34.5)	70 (16.5)
Statin	248 (31.1)	71 (45.2)	38 (36.5)	49 (43.4)	90 (21.2)
Socioeconomic parameters					
Hospital stay					
No. with available data	798	157	104	113	424
Median (IQR), d	6.0 (3.0-16.0)	13.0 (4.0-20.0)	4.0 (3.0-4.0)	17.0 (14.0-22.0)	4.0 (3.0-12.0)
Hospital charge					
No. with available data	797	157	104	113	423
Median (IQR), $	7291.3 (1879.9-16 134.1)	10 890.7 (3885.0-20 112.9)	8366.7 (6692.3-9902.1)	18 788.7 (16 515.4-23 310.0)	2730.0 (1255.3-8558.3)
Hospital parameters					
CVIT center	739 (92.2)	138 (87.9)	99 (95.2)	111 (98.2)	388 (91.5)
ACHD center	322 (40.4)	35 (22.3)	37 (35.6)	67 (59.3)	183 (43.2)
Beds, median (IQR), No.	612.0 (396.0-780.0)	460.0 (300.0-694.0)	645.5 (403.5-749.0)	612.0 (481.0-800.0)	613.0 (437.0-800.0)
Mode of hospitalization					
Emergency	263 (33.0)	126 (80.3)	6 (5.8)	5 (4.4)	126 (29.7)
No referral	128 (16.0)	58 (36.9)	10 (9.6)	10 (8.8)	50 (11.8)
Outcome parameters					
ICU care	220 (27.6)	67 (42.7)	8 (7.7)	105 (92.9)	40 (9.4)
Hospital mortality	11 (1.4)	4 (2.5)	0	2 (1.8)	5 (1.2)

Abbreviations: ACHD, adult congenital heart disease; ACS, acute coronary syndrome; BMI, body mass index (calculated as weight in kilograms divided by height in meters squared); CABG, coronary artery bypass grafting; CVIT, Japanese Association of Cardiovascular Intervention and Therapeutics; DAPT, dual antiplatelet therapy; DOAC, direct oral anticoagulant; HF, heart failure; IAPB, intra-aortic balloon pumping; ICU, intensive care unit; NOAC, novel oral anticoagulants; PCI, percutaneous coronary intervention; PCPS, percutaneous cardiopulmonary support; POBA, plain old balloon angioplasty; PTCRA, percutaneous transluminal coronary rotational atherectomy.

**Figure.**
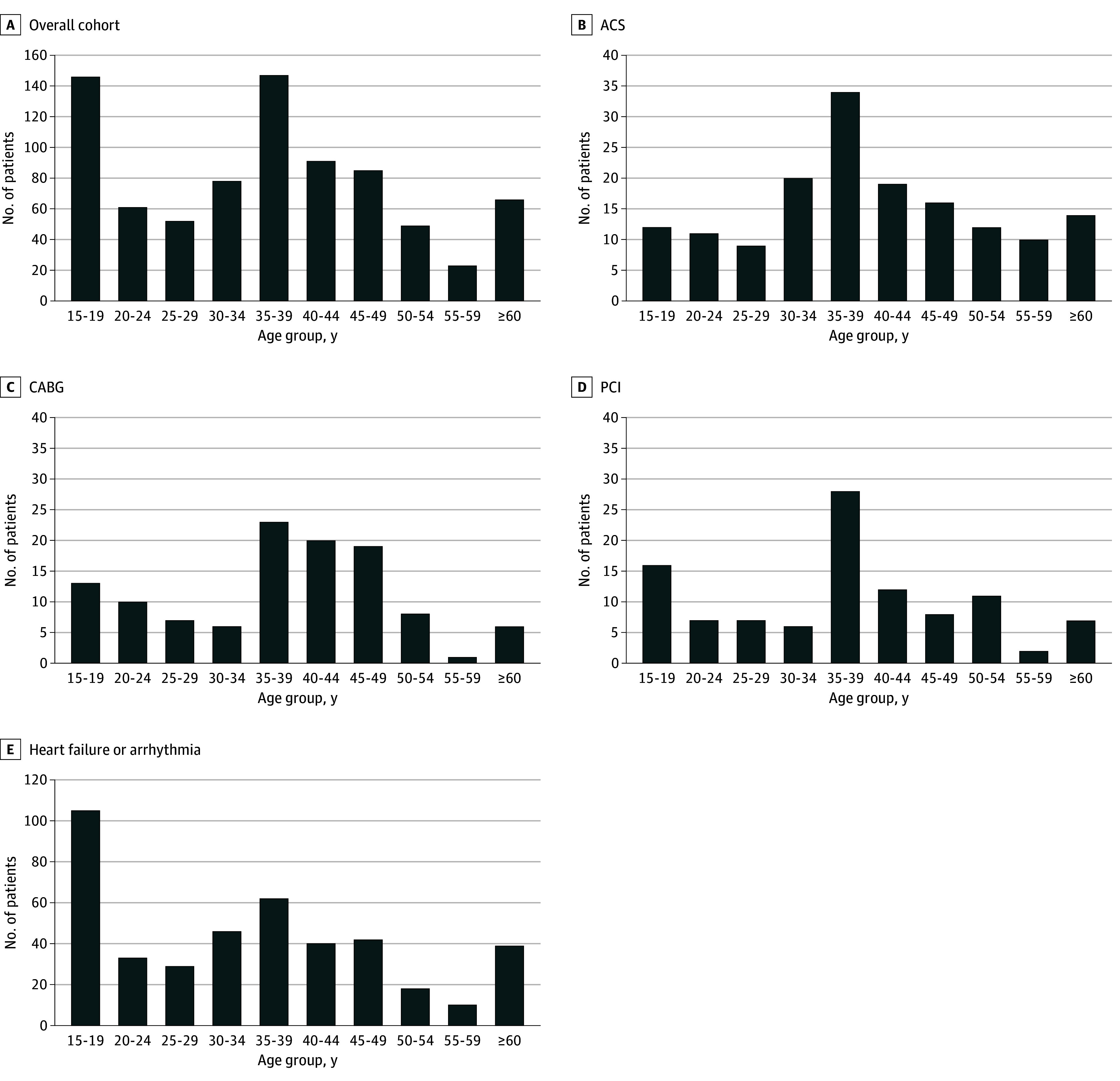
Age Distribution in the Overall Cohort, the Acute Coronary Syndrome (ACS) Group, the Coronary Artery Bypass Grafting (CABG) Group, the Percutaneous Coronary Intervention (PCI) Group, and the Heart Failure or Arrhythmia Group

## Discussion

To our knowledge, this is the first nationwide JROAD-DPC analysis of hospitalized adult patients with a history of KD. We found that severe cardiovascular events clustered in young adults without obesity, especially men, with a second surge in their late 30s. One-quarter of participants smoked, and fewer than one-third were discharged on statins, underscoring unmet opportunities for risk factor modification. Disrupted follow-up, reflected by emergency and nonreferral admissions, was independently associated with worse outcomes. Limitations include the absence of a denominator of all adult KD survivors, lack of an age-matched non-KD comparator, reliance on a single *ICD-10* code whose sensitivity and specificity for historical KD are unknown, and unavailability of outpatient data or longitudinal follow-up. Findings are therefore descriptive and hypothesis-generating but highlight critical gaps in lifelong care.

In conclusion, the present findings emphasize the critical need for structured lifelong follow-up programs, systematic health care transition strategies, and increased awareness among adult health care clinicians. Prospective registries incorporating comprehensive KD cohorts and systematic risk factor modification are warranted to clarify absolute risk and improve clinical trajectories.

## Notes

Supplement 1.
eMethods.

eReferences.

Supplement 2.
Data Sharing Statement
