# Error in Figure

**DOI:** 10.1001/jamanetworkopen.2026.6469

**Published:** 2026-03-18

In the Research Letter titled “Severe Cardiovascular Sequelae in Adults After Kawasaki Disease,”^1^ published August 12, 2025, there was an error in the Figure. In all panels, the first age group should have read 15-19. This article has been corrected.^1^
