# Supplementary material for: Severe Cardiovascular Sequelae in Adults After Kawasaki Disease
Source: JAMA Netw Open. 2025 Aug 12;8(8):e2526396. doi: 10.1001/jamanetworkopen.2025.26396 (PMC12344531; doi:10.1001/jamanetworkopen.2025.26396)
Supplement: Supplement 1. — eMethods. eReferences. [file jamanetwopen-e2526396-s001.pdf]

Supplemental Online Content

Mitani Y, Nakai M, Tsuda E, et al. Severe cardiovascular sequelae in adults after Kawasaki disease. *JAMA Netw Open.* 2025;8(8):e2526396. doi:10.1001/jamanetworkopen.2025.26396

eMethods.

eReferences.

This supplemental material has been provided by the authors to give readers additional information about their work.

© 2025 Mitani Y et al. *JAMA Network Open*.

eMethods.

Study Design and Data Source

This retrospective cohort study utilized the Japanese Registry of All Cardiac and Vascular Diseases–Diagnosis Procedure Combination (JROAD-DPC), a comprehensive nationwide claims database encompassing 1,354 Japanese Circulation Society (JCS)-certified cardiovascular hospitals across Japan, as of December 2022 [1, 2]. The JROAD-DPC database includes standardized discharge data from hospitals participating in the DPC/Per-Diem Payment System under Japan's universal health coverage. This registry captures detailed clinical and procedural information, diagnostic codes (ICD-10), hospital characteristics, patient demographics, clinical presentations (ACS, percutaneous coronary intervention [PCI], coronary artery bypass grafting [CABG], heart failure or arrhythmia [HF/ARR]), admission types (emergency, non-referral), administered treatments and in-hospital outcomes. Japanese universal health coverage was established in 1961, achieving nearly complete insurance coverage to all citizens, standardized reimbursement fees, and equitable healthcare access regardless of socioeconomic status or background [3]. Validation studies have demonstrated acceptable concordance between JROAD-DPC registry data and clinical records [4]. To assess specialist availability, we incorporated information on 749 teaching hospitals for coronary interventions certified by the Japanese Association of Cardiovascular Intervention and Therapeutics (CVIT) and 86 ACHD teaching hospitals certified by the Japanese Society for Adult Congenital Heart Disease (JSACHD), as of February, 2023 [5, 6].

Study Population and Categorization

We included adult patients (aged ≥15 years) who were hospitalized due to cardiovascular sequelae related to Kawasaki disease. Hospitalization data from April 2013 to March 2022 were analyzed. Patients were identified using the International Classification of Diseases, 10th Revision (ICD-10) code M303, documented as the primary diagnosis, admission-precipitating diagnosis, or the first or the second most resource-intensive diagnoses or comorbidities. Detailed definitions of ACS, CABG, PCI, and heart failure/arrhythmia (HF/ARR) groups are provided below In addition, the ACS group was defined by a recorded diagnosis of ACS; the CABG group was identified by the CABG procedure, excluding patients classified as ACS; the PCI group was defined by the PCI procedure, excluding those classified as ACS or CABG; and the HF/ARR group comprised patients who received treatment for heart failure or arrhythmias, excluding those already categorized in ACS, CABG, or PCI groups.

1 Acute coronary syndrome (ACS) was defined by using ICD-10 codes for acute myocardial infarction (I211.0, I21.1, I21.2, I21.3, I21.4, I21.9) and unstable angina (I200), as well as ACS (I249) recorded as 'the main diagnosis', 'the admission precipitating diagnosis', 'the first or second most

© 2025 Mitani Y et al. *JAMA Network Open*.

resource-consuming diagnosis.

2 Coronary artery bypass grafting (CABG) was determined by codes K5521, K5522, K552-21, or K552-22 (35) in the operation code 1 or 2.

3 Percutaneous coronary intervention (PCI) was identified by procedural codes for balloon angioplasty (K5461-3), stenting (K5491-3), rotavolator (K547), directional coronary atherectomy (K5481), Exima laser (K5482), intracoronary thrombolysis (K550), or aspiration thrombectomy (K550-2).

4 The procedure for heart failure treatment or arrhythmia treatment was defined by the treatment codes as follows:
In-hospital treatment for heart failure was defined by the presence of any of the following treatments: intravenous administration of catecholamines, diuretics (furosemide), vasodilators (human natriuretic peptide, milrinone, nitroglycerin), intra-aortic balloon pump, percutaneous cardiopulmonary support, ventricular assist device, or percutaneous ventricular assist device (Impella).

In-hospital treatment for arrhythmia was defined by the presence of the in-hospital administration of anti-arrhythmic compounds (class 1, II, III, IV), or aβ blocker (landiolol), treatments with the defibrillation, ablation, implantable cardioverter defibrillator or pacemaker.

Outcomes and Covariates

ICU admission was identified using specific billing codes for intensive or high care units (A301-00 or A301-02). Covariates in the analysis included patient demographics (age and sex), hospital characteristics (teaching vs. non-teaching hospital and hospital size indicated by bed count), emergency admission status, and non-referral admission status. Emergency admission was defined by code 200 (unscheduled admission), codes 3## (emergency admission), or documented ambulance utilization, while non-referral admissions were those without prior referral from any healthcare institution, including the admitting hospital itself. These variables served as indicators of disrupted patient follow-up or suboptimal outpatient management. The accuracy of primary diagnosis, procedure, and surgical records within the DPC database was previously validated [7, 8].

Ethics Statement

This study was approved by the Ethics Committees of Mie University Hospital (approval numbers H2022-201, H2022-001) and the National Cerebral and Cardiovascular Center (approval number R20021-4). Informed consent was waived due to the retrospective analysis of anonymized data

© 2025 Mitani Y et al. *JAMA Network Open*.

under the "opt-out" principle.

eReferences

1. Yasuda S, Miyamoto Y, Ogawa H. Current Status of Cardiovascular Medicine in the Aging Society of Japan. *Circulation*. Sep 4 2018;138(10):965-967. doi:10.1161/CIRCULATIONAHA.118.035858

2. Nishi M, Miyamoto Y, Iwanaga Y, et al. Hospitalized Patients, Treatments, and Quality of Care for Cardiovascular Diseases in Japan - Outline of the Nationwide JROAD Investigation. *Circ J*. Nov 19 2024;doi:10.1253/circj.CJ-24-0704

3. Ikegami N, Yoo BK, Hashimoto H, et al. Japanese universal health coverage: evolution, achievements, and challenges. *Lancet*. Sep 17 2011;378(9796):1106-15. doi:10.1016/S0140-6736(11)60828-3

4. Nakai M, Iwanaga Y, Sumita Y, et al. Validation of Acute Myocardial Infarction and Heart Failure Diagnoses in Hospitalized Patients With the Nationwide Claim-Based JROAD-DPC Database. *Circ Rep*. Feb 20 2021;3(3):131-136. doi:10.1253/circrep.CR-21-0004

5. Certified teaching hospitals of Japanese Association of Cardiovascular Intervention and Therapeutics. Japanese Association of Cardiovascular Intervention and Therapeutics website. https://www.cvit.jp/_new/certification/. Accessed March 1, 2025.

6. Certified teaching hospitals of Japanese Society for Adult Congenital Heart Disease, Japanese Society for Adult Congenital Heart Disease website. https://www.jsachd.org/specialist/list-facility/. Accessed March 1.2025

7. Yamana H, Moriwaki M, Horiguchi H, Kodan M, Fushimi K, Yasunaga H. Validity of diagnoses, procedures, and laboratory data in Japanese administrative data. *J Epidemiol*. Oct 2017;27(10):476-482. doi:10.1016/j.je.2016.09.009

8. Konishi T, Yoshimoto T, Fujiogi M, et al. Validity of operative information in Japanese administrative data: a chart review-based analysis of 1221 cases at a single institution. *Surg Today*. Oct 2022;52(10):1484-1490. doi:10.1007/s00595-022-02521-8

© 2025 Mitani Y et al. *JAMA Network Open*.
