# Supplementary material for: Severe Cardiovascular Sequelae in Adults After Kawasaki Disease
Source: JAMA Netw Open. 2025 Aug 12;8(8):e2526396. doi: 10.1001/jamanetworkopen.2025.26396 (PMC12344531; doi:10.1001/jamanetworkopen.2025.26396)
Supplement: Supplement 2. — Data Sharing Statement [file jamanetwopen-e2526396-s002.pdf]

Data Sharing Statement

Mitani. Severe Cardiovascular Sequelae in Adults After Kawasaki Disease. *JAMA Netw Open*. Published August 12, 2025. doi:10.1001/jamanetworkopen.2025.26396

Data
Data available: No

Additional Information
Explanation for why data not available: individual patient data and a data dictionary is prohibited by the regulation of the data management agent
